# Editorial: Stem cell technologies meet stem cell biology to shine new light into tropical infectious diseases

**DOI:** 10.3389/fcimb.2024.1411728

**Published:** 2024-05-07

**Authors:** Alena Pance, Gabriel Rinaldi

**Affiliations:** ^1^ School of Life and Medical Sciences, University of Hertfordshire, Hatfield, United Kingdom; ^2^ Department of Life Sciences, Aberystwyth University, Aberystwyth, United Kingdom

**Keywords:** embryonic stem cells, induced pluripotent stem cells, organoids, parasite stem cells, tropical infectious diseases, protozoan parasites, metazoan parasites, vectors

Innovative technologies have revolutionized fundamental and translational biomedical research over the last decade. The increasing use of Embryonic Stem Cells and the reprogramming of Induced Pluripotent Stem Cells (iPSCs), in tandem with the advent of genome editing, have given access to cell types, cellular processes and molecular mechanisms that had previously been out of reach. Particularly in the field of parasitic infectious diseases, culture systems that replicate natural niches where these pathogens dwell are extremely difficult to develop in the laboratory. Continuous access to primary cell samples is challenging; cell types involved in host-parasite interactions are extremely difficult to maintain *in vitro* or *ex vivo* and many other challenges hinder progress in understanding the biology of pathogens, including their development and interaction with hosts. The opportunities offered by these cutting-edge technologies in this field are extraordinary, though not without their own hurdles to overcome. These include reliable differentiation protocols to generate high-quality model systems, and development of novel assays to assess parasite invasion, host interaction, immune evasion, and infection establishment. Moreover, the perception and acceptance of these model systems within the scientific community also represents a challenge. Tackling all these elements will pave the way towards the discovery of novel control strategies for infectious diseases. In this Special Research Topic, we examine recent developments and applications of Stem Cell technologies and further explore new approaches that will enrich and widen the horizon for understanding tropical infectious diseases. Furthermore, the biology of recently described Stem Cell systems in complex metazoan parasites, which are being revealed through cellular, molecular, and ‘omics’ approaches, is discussed. Insights into toti- and pluri-potent cells of parasites critical for their development and host interaction will unveil targets for novel diagnostic and control strategies.

Over half of the world’s population, particularly in the most impoverished regions of the globe, are at risk of infection with two major groups of parasites. These organisms are the main focus of this Research Topic: protozoa and metazoa parasites, including apicomplexa and helminths, respectively. Here, we have assembled 12 articles that contribute to this exciting and thriving field.

Malaria is one of the first infectious diseases to which this technology has been applied. Two cell types are mainly involved in this disease: hepatocytes, which are inaccessible for experimental studies, and erythrocytes, which are genetically elusive because they are anucleated. The opportunity to manipulate erythrocytes is described by Satchwell in his analysis of how Stem Cell technologies can help unravel the mechanisms underlying the erythrocytic life cycle of malaria parasites. It is also clearly highlighted by Pance et al., who show that erythrocytic cells can be derived from a variety of Stem Cells, including patient-reprogrammed iPSCs. This allows modification using site-specific genome editing as well as preserving specific genomic backgrounds. This approach not only facilitates access to a specific cell type, the natural niche of the parasite, but also facilitates the study of the impact of specific human proteins on the infection and disease progression. In this line, a novel development is introduced by Parres-Mercader et al. which envisions the generation of 2D and 3D culture organoid systems that mimic the mosquito midgut and salivary gland environments to study the intra-vector developmental stages of malaria parasites. Outcomes of these approaches will shine light on to strategies for transmission control.

The pathogenic agent of the American Trypanosomiasis or Chagas Disease, *Trypanosoma cruzi*, is one of the organisms that has long lacked adequate culturing systems, as it preferentially invades cardiac muscle. The use of Stem Cells has facilitated the production of cardiomyocytes *in-vitro*, providing a natural environment, and enabling the use of ‘omics’ approaches to study the infection, as shown by Venturini et al. A time course experiment of the infection revealed activation of immunity-related genes and demonstrated that the parasite exploits cardiomyocyte stress response and inflammation to establish the infection. Excitingly, the possibility of disease-specific responses has been explored by Oliveira et al. using iPS-derived cardiomyocytes generated from patients with chronic and asymptomatic disease. This study reveals significant transcriptomic differences between the two disease states and starts unraveling molecular mechanisms underlying cell damage in chronic cardiomyopathy. Moreover, the authors hypothesize that the immune response may contribute to a milder pathogenesis. In addition, the poorly explored role of the intestinal tissue infection during the oral transmission of trypanosomes and development of the chronic disease is moving within reach by using murine colon organoids as presented by Daghero et al. Successful invasion and replication was achieved in these 2D and 3D cellular structures, showing marked differences with cultures using intestinal cell lines, reflecting a more natural environment. Finally, while *in-vitro* systems to study intra-vector developmental stages of trypanosomes have not yet taken advantage of Stem Cell technologies, Smircich et al. pointed out the potential contribution of 3D insect-derived culture systems in this neglected field.

The emergence of 2D and 3D culture systems providing specific intestinal models to characterize the life and sexual stages of *Toxoplasma gondii* in the enteric epithelium is comprehensibly explored by Sena et al. This Review of novel experimental platforms being developed highlights important challenges and opportunities to advance this field in the near future. The exciting prospects of these cellular systems to dissect the molecular mechanisms underlying the impact of *T. gondii* infection on pregnancy are also analyzed and discussed by Faral-Tello et al., paving the way to a better understanding of the infection in the maternal-foetal interface and hence revealing targets for novel control strategies.

Metazoan parasites, such as helminths, rely on complex Stem Cell systems to develop throughout their complex life cycles where the parasite metamorphosizes across different body plans. These systems represent a continuous source of differentiated cells that facilitate healing of parasite tissues that are exposed to adverse conditions within the host. Some of these cells underlie the tumor-like growth of the metacestode stage of the tapeworm *Echinococcus multilocularis* within host organs. Using ‘omics’ approaches, Herz et al. identified >1,100 genes associated with germinative cells, some of which were validated and characterized by *in situ* hybridization and pulse-chase experiments. Furthermore, the authors characterized the transcriptomic profile of primary cell cultures derived from whole parasites, initiating the understanding of molecular mechanisms that would advance the establishment of helminth-derived cell lines for the first time. In a similar fashion, but using the model tapeworm *Hymenolepis microstoma*, Montagne et al. explored cell proliferation and differentiation during the parasite’s development, employing a combination of molecular and imaging approaches. This first comprehensive dissection of a model parasite development provides a platform for equivalent studies on helminths that infect humans and animals. The free-living nematode *Caenorhabditis elegans* also represents an extremely useful model for which ‘bulk’ and single-cell omics-based datasets coupled with functional genomics tools are available. Davies et al. reviewed the latest studies on the molecular mechanisms employed by the bacterium *Pasteuria* spp. for specific host recognition. The development of microbial biocontrol products including *Pasteuria* to specifically infect root-knot nematodes promises novel control avenues. The authors discussed key findings involving worm Stem Cell-like cells, named Seam Cells that appear to play a critical role in the recognition of the parasite by the bacteria.

The broad collection of articles in this Research Topic, >50% of which originated from endemic countries, shows the importance of Stem Cell-based approaches to understand some of the most devastating diseases affecting humans and animals worldwide. The development of culture systems recapitulating definitive host and vector conditions and the opportunities to study patient-specific effects and human polymorphisms as well as the role of parasite Stem Cells offer unlimited potential to unravel biology and discover new avenues for prevention and treatment (**Figure 1**). We look forward to future developments of Stem Cell tools and their combination with genome editing and single cell omics studies to drive and advance this fascinating and promising field.

**Figure 1:**
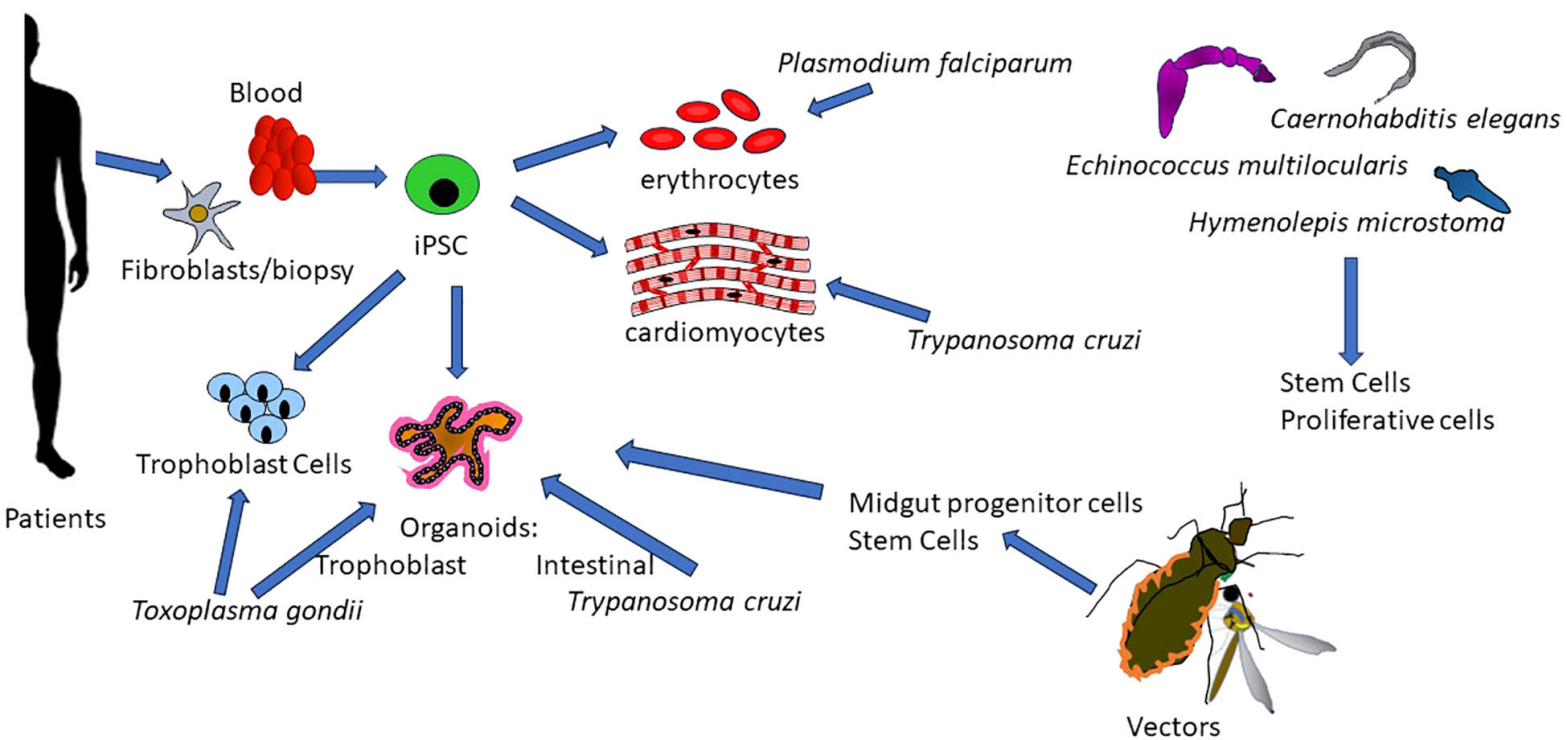
Stem Cell-based approaches to study tropical infectious diseases. Induced Pluripotent Stem Cell lines (iPSC) can be derived from blood cells or fibroblasts. These iPSCs can be cultured and stored indefinitely and differentiated into different cell types relevant to the study of parasites: erythrocytes, cardiomyocytes, or trophoblasts. Organoids can also be generated from iPSCs to provide tridimensional niches mimicking the natural environments where these parasites dwell. The potential for replicating these advances in the vectors involved in the transmission of these diseases is also being developed. Understanding of stem and proliferative cells in multicellular metazoan parasites is a new field, leading to better knowledge of the biology of these parasites and hence revealing targets for novel control strategies.

## Author contributions

AP: Conceptualization, Writing – original draft, Writing – review & editing. GR: Conceptualization, Writing – original draft, Writing – review & editing.

